# Genome sequence of freshwater sediment isolated fungus *Westerdykella aurantiaca* strain NNIBRFG27121

**DOI:** 10.1128/MRA.00613-23

**Published:** 2023-11-08

**Authors:** Ki-Tae Kim, Jea Hyeoung Kim, Yoeguang Hue, Haejun Jeong, Hyeon Jung Hwang, Li Ra Yoo, Wonsu Cheon, Jaeduk Goh

**Affiliations:** 1 Department of Agricultural Life Science, Sunchon National University, Suncheon, South Korea; 2 Department of Plant Medicine, Sunchon National University, Suncheon, South Korea; 3 Fungal Resources Research Division, Nakdonggang National Institute of Biological Resources, Sangju, South Korea; University of California, Riverside, Riverside, California, USA

**Keywords:** *Westerdykella aurantiaca*, fungus, freshwater, sediment, genome

## Abstract

We sequenced the genome of *Westerdykella aurantiaca* NNIBRFG27121 strain isolated from the wetland of Maehwamarum Habitat in Korea. The final assembly consisted of six scaffolds with a size of 31.96 Mb and an N50 of 8,770,400 bp. This genome will help in comparing species within the *Westerdykella* genus.

## ANNOUNCEMENT

The *Westerdykella* genus, belonging to Sporormiaceae, has been identified as a potential bioresource for producing various bioactive substances (1). However, limited genomic resources have restricted the exploration of its full potential (2) (Table 1). Therefore, we sequenced the genome of *Westerdykella aurantiaca* strain NIBRFG27121 to enhance the further comparative genomic studies of the genus.

The fungal strain was isolated from the sediments in Maehwamarum Habitat wetland in Incheon (37°38′6″N, 126°31′47″E), Korea, in September 2019. Sediment in the wetland was collected by garden trowel and transported to the laboratory with freshwater in an icebox. As previously described, the fungal strain used in this study was isolated by a dilution method and identified as *W. aurantiaca* using molecular phylogenetic analyses of rDNA and beta-tubulin sequences (3). The isolate was deposited in Freshwater Bioresources Culture Collection (FBCC) of Nakdonggang National Institute of Biological Resources, South Korea, under FBCC-F1472. The isolate was cultured for 7 days at 25°C on potato dextrose agar (PDA; BD, Franklin Lakes, NJ, USA) for DNA extraction. Young mycelia on PDA were transferred to potato dextrose broth (BD) and cultured for 7 days at 25℃ without light with 150 rpm in a shaking incubator. The mycelia were then harvested using a membrane filter, and DNeasy plant mini kit (Qiagen, Hilden, Germany) was used according to the manufacturer’s manual. The quantity (39.4 µg) and quality of DNA were checked using PicoGreen dsDNA Assay Kit (Invitrogen, Waltham, MA, USA) with Victor three fluorometry (PerkinElmer, Waltham, MA, USA) and gel electrophoresis method.

**TABLE 1:** Genome assembly statistics of *Westerdykella* species

Species	*Westerdykella aurantiaca*	*Westerdykella ornata*	*Westerdykella* sp.
Strain	NIBRFG27121	CBS 379.55	P71
Genome assembly size (Mbp)	31.95	26.98	31.80
No. of scaffolds	6	94	1,985
N50 (bp)	8,770,400	803,790	61,894
L50	2	10	145
GC (%)	51.91	52.89	52.92
Sequencing technology	PacBio, Illumina	Illumina	Illumina
Whole Genome Seq. Project	JASJFJ01	JAAEJA01	JAFHBW01
Isolated location	Freshwater sediments, Korea	Mangrove mud, Mozambique	*Persicaria acuminata*, Brazil
Reference	This study	(2)	Senabio et al., 2021 (unpublished)

A hybrid assembly strategy using PacBio and Illumina platforms was applied for the genome of *W. aurantiaca*. The long-read library was generated using SMRTbell Express Templates Library kit, and its quality was controlled using Femto Pulse System and 2100 Bioanalyzer System (Agilent Technologies, Santa Clara, CA, USA). For the short-read library, TruSeq Nano DNA Library Prep Kit was used according to the user manual, and the quantity of library was checked using PicoGreen dsDNA Assay Kit and D1000 TapeStation System (Agilent Technologies). The long-read sequencing and the paired-end short-read (150 bp) sequencing were performed using PacBio Sequel-I and Illumina HiSeqXten platforms (Macrogen, Seoul, South Korea), respectively.

The raw subread data from the long-read sequencing were filtered by SMRTLink v.10.1.0.119588 and produced 1,896,089 subreads (21,118, 824,046 nt) with an N50 read length of 16,672 nt. On the other hand, the short-read sequencing with quality control using in-house script and Trimmomatic v0.38 (4) produced a total of 25,080,350 reads with 3,783,406,606 nt. The long-read sequences were assembled into a genome using HGAP v.4.0 pipeline with default parameters (5), followed by short-read error correction with Pilon v.1.22 (6). The genome of *W. aurantiaca* strain NNIBRFG27121 comprised six scaffolds with a size of 31.96 Mb, a GC content of 51.9%, and an N50 of 8,770,400 bp (Table 1). To validate the assembly accuracy, the filtered Illumina short reads were mapped back to the assembled genome using BWA v.0.7.17 (7). In consequence, 94.67% of reads were mapped with 99.96% coverage. Finally, the genome assembly was checked using BUSCO v.5.1.3 against the eukaryota_odb10 data set (8). Of the total of 255 BUSCO data set searched, the assembly displayed 248 complete BUSCOs (approximately 97.25%), with 247 single copies (96.86%). This high-quality genome of *W. aurantiaca* will be valuable for further comparative genomic studies on *Westerdykella*.

## Data Availability

BioProject and BioSample records for the *Westerdykella aurantiaca* genome were created in the National Center for Biotechnology Information database with accession numbers PRJNA973833 and SAMN35152317, respectively. The raw data were submitted to Sequence Read Archive with accession numbers SRR25009817 (PacBio) and SRR25133175 (Illumina). Finally, the genome assembly was submitted to GenBank with Whole Genome Sequencing (WGS) accession number JASJFJ000000000.
